# An Efficient and Safe Rapid Aging Technology for Tea: UV-C Irradiation Enhances the Taste and Aroma of Fresh Pu’er Raw Tea Toward a Naturally Aged Profile

**DOI:** 10.3390/plants14121851

**Published:** 2025-06-16

**Authors:** Xinghai Zhang, Xinyu Feng, Yani Pan, Hanfei Wuzeng, Xinxin Wang, Anran Yan, Lin Xiang, Yaping Lin, Ping Chen, Qiang Chu, Liping Liu

**Affiliations:** 1 International Tea Culture College, Zhejiang Shuren University, Hangzhou 310015, China; 2 Tea Research Institute, College of Agriculture and Biotechnology, Zhejiang University, Hangzhou 310058, China; 3180100289@zju.edu.cn (X.F.); yanipan@zju.edu.cn (Y.P.); 22316146@zju.edu.cn (X.W.); 12416100@zju.edu.cn (A.Y.); 12416092@zju.edu.cn (L.X.); pingchen@zju.edu.cn (P.C.); 3 College of Tea and Food Science, Wuyi University, Wuyishan 354300, China; 4 Huzhou Key Laboratory of Innovation and Application of Agricultural Germplasm Resources, Huzhou Academy of Agricultural Sciences, Huzhou 313001, China

**Keywords:** rapid aging, UV-C irradiation, Pu’er raw tea, aroma, taste

## Abstract

Pu’er raw tea, a representative tea with a positive correlation between quality and storage time, has a unique aging process. It can not only reduce the heavy astringent taste of newly produced tea, but also has a complex and fragrant aging aroma. However, the extremely slow natural aging process often takes years or even decades for quality transformation, along with the risk of termite infestation, odor absorption, etc. This study found that UV-C irradiation could significantly accelerate the aging process of tea. It enhanced the richness of the aroma, while reducing astringency and creating a smoother and mellower taste. The results of substances analysis revealed an increase in key aroma compounds such as alcohols, aldehydes and terpenes, and a decrease in catechin and caffeine with UV-C irradiation, which is consistent with the natural aging trend. An efficient and safe rapid aging technology of Pu’er raw tea has been successfully established.

## 1. Introduction

Pu’er raw tea, a renowned tea native to Yunnan, is highly favored for its unique flavor and aging potential [1]. According to the national standard for Pu’er tea [2], it is made from sun-dried large-leaf tea produced in protected geographical areas of Yunnan and classified into two types: raw and ripe Pu’er tea. Pu’er raw tea is characterized by its distinctive bitterness and astringency, similar to green tea [3]. However, as time passes and natural aging occurs, the astringency diminishes, resulting in a mellower, more harmonious flavor with increased complexity and a more mature aroma. Yet, the natural aging process is extremely slow, typically taking years or even decades to achieve the desired quality [4]. The long-term storage process incurs significant financial costs and increases risks like termite infestation, exposure to strong light, and absorption of off-flavors, which can degrade the tea’s quality [5]. Thus, there is an urgent need for a safe and efficient method to accelerate the aging process of high-quality Pu’er raw tea. Furthermore, other types of tea, such as white tea, also demonstrate the characteristic of “improving with age,” highlighting the broad prospects for research into rapid aging technologies [6].

Currently, rapid aging methods such as 60Co-γ irradiation, high-pressure pulse treatment, and control of temperature and humidity have their pros and cons, respectively. 60Co-γ irradiation rapidly accelerates oxidation reactions in the tea through radiation, simulating the effects of years of natural aging [7,8]. However, this method raises concerns about safety and potential nutritional changes in the tea [7]. High-pressure pulse treatment accelerates internal chemical reactions in tea without compromising its nutritional content. While high-pressure processing preserves the original color, aroma, and flavor of tea and effectively shortens aging time, its complexity and high equipment costs limit its market adoption [8]. The control of temperature and humidity could simulate natural aging conditions well, although this also takes a long time and the aging effect is uncertain. UV-C irradiation has a strong oxidation effect, which could accelerate the chemical oxidation reaction in the aging process in a short time [9]. It has been successfully applied to fruits like strawberries and pepino fruit to extend shelf life or improve the sensory quality [10,11]. The application of UV-C irradiation in tea is quite limited, and only a few researchers have used UV-C irradiation to replace the heat-pasteurization of green tea beverages [12]. UV-C irradiation technology shows great potential in accelerating the aging of tea.

This study aims to investigate the effects of UV-C irradiation on the quality of Pu’er raw tea and establish a rapid aging method. A specially designed UV-C irradiation chamber was used to achieve rapid aging of Pu’er raw tea. The impact of UV-C irradiation on sensory qualities was assessed through sensory evaluation and quantitative descriptive analysis, to visually determine its similarity to naturally aged samples. Gas chromatography–mass spectrometry (GC-MS) was employed to elucidate the effects of UV-C irradiation on the volatile aroma compounds of Pu’er raw tea. High-performance liquid chromatography (HPLC) was utilized to clarify the changing trends in non-volatile substances with UV-C irradiation. Multivariate statistical analysis was applied to determine the group differences between different UV-C irradiation durations and naturally aged samples. The objective was to evaluate the potential of UV-C irradiation as a rapid aging method for Pu’er raw tea and screen for optimal UV-C treatment conditions. This study demonstrates that UV-C treatment significantly accelerates the aging process of Pu’er raw tea, achieving the chemical changes required for natural aging in a shorter time frame. This study successfully constructed an efficient, rapid, and safe aging technology for Pu’er raw tea, providing theoretical and practical support for the industrialized rapid production of tea. It could potentially be extended to other types of tea, enhancing their market competitiveness, and offering a new technological pathway for producing storage-resistant teas.

## 2. Materials and Methods

### 2.1. Preparation of Tea Samples and Chemicals

The fresh and naturally aged Pu’er raw tea samples originated from Shuanghe Hot Spring, Anding Town, Jingdong Yi Autonomous County, Pu’er City, Yunnan Province. They were all processed using the same Pu’er raw tea processing techniques by Pu’er Tianze Tea Industry Co., Ltd, Pu’er, China. Gallic acid, 8 types of catechin monomers, and 9 types of flavonoid glycoside monomers were purchased from Shanghai Yuanye Bio-Technology Co., Ltd, Shanghai, China. Acetonitrile, acetic acid, methanol, and formic acid (chromatographic grade) were obtained from Aladdin Reagent Co., Ltd., Shanghai, China. Potassium dihydrogen phosphate, disodium hydrogen phosphate, phosphoric acid, boric acid, sodium hydroxide, *O*-phthalaldehyde, thiopropionic acid, and ethanol (analytical grade) were supplied by China National Pharmaceutical Group Chemical Reagent Co., Ltd, Beijing, China. Ultrapure water was filtered using a Millipore ultrapure water system throughout the experiment. The LC-20A high-performance liquid chromatography system with RF-20A/SPD-M20A detectors was from Shimadzu, Japan [13]. The ACQUITY UPLC ultra-performance liquid chromatography-mass spectrometry system was from Waters, MA, USA. The HWS28 electric constant temperature water bath was from Shanghai Yiheng Scientific Instrument Co., Ltd., Shanghai, China, and the Micro CL21R microcentrifuge was bought from Thermo Scientific, Baden-Württemberg, Germany.

### 2.2. Treatment of Tea Samples Using UV-C Irradiator

Pu’er raw tea samples were subjected to UV-C irradiation using a custom-designed UV-C irradiator to investigate the effects of different exposure durations on the aging process. The irradiator consists of an opaque, sealed box with controllable UV-C lamps mounted on the inside of the top lid. The UV-C lamps emitted light at a wavelength of 254 nm, which is commonly used to induce oxidative changes in organic materials. The distance between the UV-C lamps and the tea samples was set at 30 cm to ensure optimal irradiation intensity while avoiding excessive heat or damage to the tea leaves. During the experiment, fresh Pu’er raw tea samples were spread evenly across the bottom of the box, ensuring that the leaves did not overlap each other. This arrangement was crucial to ensure that all tea samples received uniform UV-C irradiation. The tea samples were divided into six groups based on the duration of UV-C exposure: 0 h (blank control), 1 h, 4 h, 6 h, 8 h, and 10 h. The naturally aged Pu’er raw tea (pw), was included as a reference to compare the effects of UV-C treatment with traditional aging. The naturally aged samples had been stored under controlled conditions for 5 years, allowing for the natural development of the chemical and sensory characteristics typical of aged Pu’er raw tea. Each group of tea samples was exposed to UV-C irradiation in a temperature-controlled environment (25 °C ± 2 °C) to minimize the influence of external factors on the aging process. The relative humidity during the treatment was maintained at 45–55% to simulate the typical storage conditions of Pu’er raw tea. After the designated irradiation period, the tea samples were immediately removed from the irradiator and allowed to cool to room temperature. The samples were then sealed in airtight containers to prevent any further oxidation or environmental exposure until further analysis.

### 2.3. Sensory Quality Assessment and Colorimeter Measurement

The sensory quality assessment was conducted according to the guidelines of the “Tea Sensory Review Methods” [14] at room temperature (23 °C ± 1 °C). Specifically, each dry tea sample weighing 3 g was placed in a standard 150 mL column cup and brewed with boiling water for 5 min. Subsequently, ten expert panel members (five males and five females) independently evaluated the taste characteristics of the tea infusion (at a temperature of 60–70 °C) [15]. The panel also assessed the aroma of the tea residues after draining the infusion. To maintain consistent temperature, containers with tea residues were kept in a 50 °C water bath. Each assessor provided individual comments and scores based on their expert analysis. A series of specific aroma descriptors were applied, including pekoe, floral, clean, and sweet. Similarly, taste descriptors such as clean, fresh, heavy, mellow, and sweet were used for characterization. The rating scale ranged from 0 to 10, with 0 indicating the absence or imperceptible intensity of a particular characteristic, and 10 representing an exceptionally high intensity. Parallel trials were conducted three times to ensure the reliability and consistency of the assessments.

Furthermore, the color of the tea infusion was assessed using a colorimeter, with the L*, a*, b*, c*, and h* values employed for precise color measurement [16]. The L* value represents the luminosity variation, extending from black to white, where higher values indicate lighter shades (+white) and lower values indicate darker shades (−black). The a* value measures the contrast between red and green hues, with positive values indicating a shift towards red (+red) and negative values indicating a shift towards green (−green). Similarly, the b* value reflects the difference between yellow and blue hues, where positive values denote a tendency towards yellow (+yellow) and negative values denote a tendency towards blue (−blue). The c* value, or chroma, indicates the color’s purity or saturation, with higher values representing more vivid colors. The h* value, or hue angle, specifies the specific color tone, measured in degrees, providing a precise indication of the color’s position on the color wheel. To guarantee precision, each tea sample’s color measurement was conducted three times.

### 2.4. GC-MS Detection and Analysis

A HP-5MS capillary column (30 m × 0.25 μm × 0.25 mm) was used for gas chromatography [17]. High-purity helium (purity 99.999%) served as the carrier gas, with a constant flow rate of 1.0 mL/min. Splitless injection was employed. The initial temperature was set at 50 °C and maintained for 5 min, followed by an increase to 210 °C at a rate of 3 °C/min, where it was held for 5 min. The temperature was then raised to 230 °C at a rate of 15 °C/min and held for 5 min. The interface temperature was set at 230 °C, and the ion source temperature at 250 °C. The ionization mode was electron impact (EI), with an electron energy of 70 eV. The mass scan range was 35–450 amu. Total ion current (TIC) peak integration was performed on the mass spectra. Each peak was identified using the NIST spectral library, and aroma components were determined based on a similarity index of over 75%, in conjunction with reference materials, the literature, and retention times.

### 2.5. HPLC Analysis

The quantitative analysis of catechins and alkaloids in Pu’er raw tea samples was performed using HPLC-UV detection method and external standardization [18]. The specific operating conditions were as follows: agilent TC-C18 column (4.6 mm × 250 mm, 3.5 µm), mobile phase A was a mixture of water, acetonitrile, and acetic acid (V:V = 193:6:1), mobile phase B was a mixture of water, acetonitrile, and acetic acid (V:V = 139:60:1). The gradient elution program was as follows: 75% B (0~40 min), 75%~20% B (40~45 min), 20% B (45~50 min). Flow rate was 1 mL/min, detection wavelength was 280 nm, and column temperature was 25 °C [19].

### 2.6. Statistical Analysis

Principal Component Analysis (PCA) and Orthogonal Partial Least Squares Discriminant Analysis (OPLS-DA) were performed using SIMCA-P 14.1 software to assess the relationships between different treatment groups and explore underlying patterns in the chemical composition and sensory data [20]. Heatmaps and hierarchical cluster analyses were generated using TB Tools, while histograms were plotted using GraphPad Prism 8.0 software, which was selected for its capability to generate high-quality, publication-ready figures. Statistical analysis, including one-way analysis of variance (ANOVA), was conducted using IBM SPSS Statistics 26 software. The *p*-value of less than 0.05 was considered to be significant.

## 3. Results and Discussion

### 3.1. The Impact of UV-C Treatment on the Sensory Characteristics of Pu’er Raw Tea

**Figure 1:**
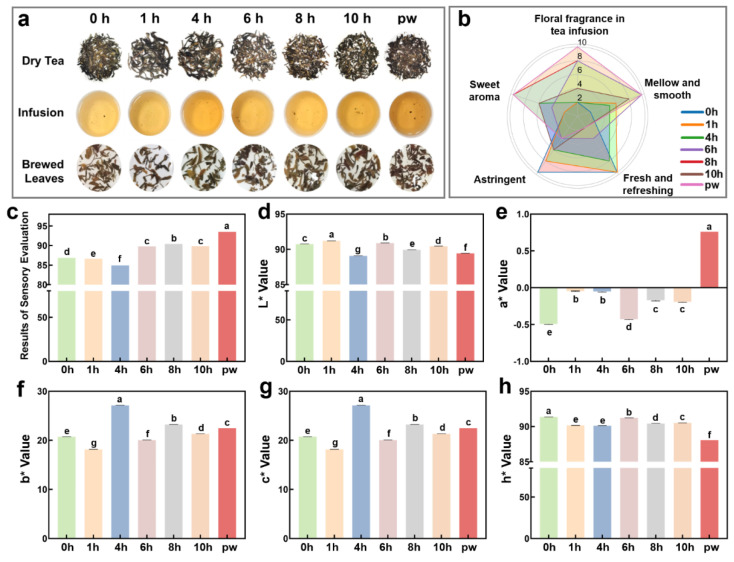
Effect of UV-C treatment on sensory properties of Pu’er raw tea. (**a**) Appearance of dry tea, tea infusions and leaf residue of Pu’er raw tea samples. (**b**) Sensory scores of taste and aroma descriptors. (**c**) Sensory quality scores. (**d**–**h**) The (**d**) L* value, (**e**) a* value, (**f**) b* value, (**g**) c* value, and (**h**) h* value of Pu’er raw tea samples. Groups without any same letters labeled were significantly different (*p* < 0.05).

This study systematically investigated the effects of UV-C irradiation on the sensory characteristics of Pu’er raw tea and compared them with naturally aged samples. Different UV-C irradiation time groups were established and naturally aged Pu’er raw tea (aged for five years) was set as the control group. During the experiment, the sensory characteristics of the samples were evaluated in terms of appearance, aroma, taste, and infused leaf condition. In the fresh Pu’er raw tea samples, the appearance was characterized by a regular cake shape, with a tight and compact surface and tight, well-defined leaves. The color was bright green or yellow-green. The aroma was primarily fresh, accompanied by slight floral notes (Figure 1a, Table 1). However, the taste was brisk with a pronounced astringency. Compared to the naturally aged tea samples (aged for five years), the fresh tea lacked complex aromas such as aged, woody, or medicinal notes, resulting in a less layered aroma profile and a relatively simple taste. This is one of the reasons why fresh Pu’er raw tea is less favored in the market compared to aged tea. As the duration of UV-C irradiation increased, the aroma and taste characteristics of the samples gradually changed. According to the data from the figures, the aroma of the samples transformed from fresh to sweet as the irradiation time increased from 0 h to 8 h, eventually developing a honey-like aroma (Figure 1b). Specifically, the aroma of the samples in the 4 h and 6 h groups transitioned from freshness to sweetness, and floral and fruity notes began to appear in the tea infusion. After 8 h of irradiation, the aroma profile of the samples more closely resembled that of naturally aged tea, with floral, sweet, and distinct aged notes. This trend suggests that UV-C irradiation accelerated oxidation and the transformation of volatile compounds in the tea. In terms of taste, UV-C irradiation also had a significant effect on the smoothness of the samples. The study found that with longer UV-C exposure, the astringency of the samples gradually decreased, especially in the 6 h and 8 h groups, where the smoothness of the tea infusion markedly improved (Figure 1b).

Based on the sensory evaluation scores (Figure 1c), the samples treated with 8 h of UV-C irradiation achieved scores second only to those of the naturally aged tea (aged for five years), indicating that this treatment duration can replicate the flavor characteristics of long-term aging within a short period. In contrast, UV-C irradiation exceeding 8 h led to a lighter tea infusion color, increased brightness, and reduced chroma saturation (Figure 1d–h), which may be due to over-oxidation and a decline in sensory quality. These results suggest that UV-C irradiation should not exceed 8 h, as this is the optimal duration for achieving the best aging effect. Moreover, the experiment further demonstrated the impact of UV-C treatment on the color of the tea infusion through chromatic analysis. Figure 1b shows that the L value (lightness) of the 10 h irradiation group was the highest, significantly exceeding that of both the 8 h group and the control group, indicating that prolonged UV-C treatment increases the brightness of the tea infusion. However, the decreases in the a and b values (representing redness and yellowness, respectively) suggest a reduction in color saturation, implying that excessive UV-C irradiation may result in diminished tea quality. Overall, an 8 h UV-C treatment duration not only maintains stable color characteristics of the tea infusion but also achieves ideal results in terms of aroma and taste.

### 3.2. Analysis of Aroma Components of Pu’er Raw Tea Treated with UV-C for Different Duration

**Figure 2:**
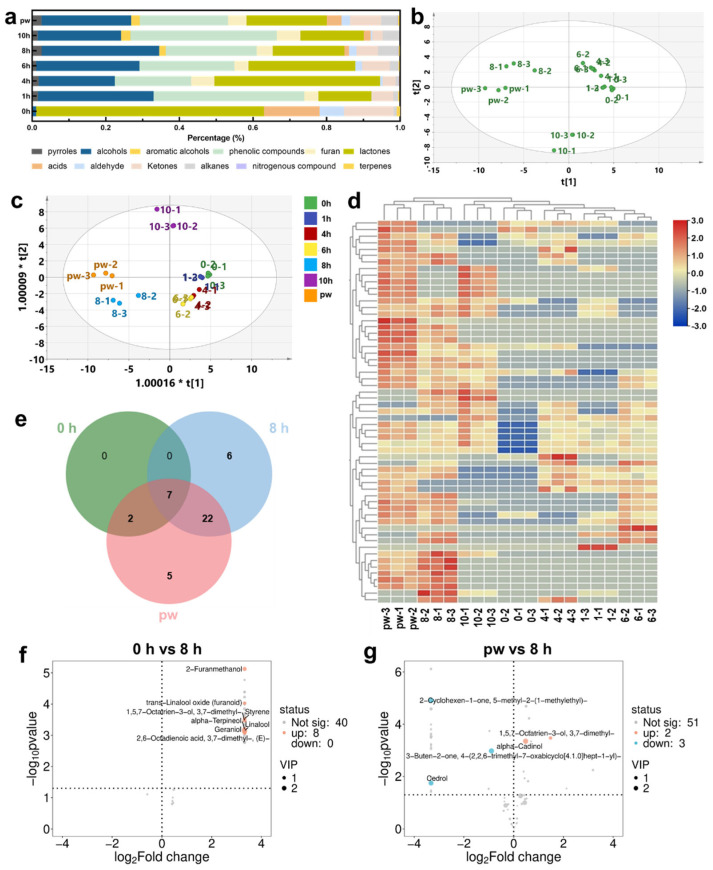
Analysis of aroma substances in Pu’er raw tea samples treated with UV-C for different durations. (**a**) The percentage distribution of various compounds in Pu’er raw tea with or without UV-C irradiation. (**b**,**c**) The (**b**) PCA and (**c**) OPLS-DA analysis of Pu’er raw tea samples treated with UV-C for different durations. (**d**) Relative abundance heatmaps of key volatile compounds. (**e**) Venn diagram of volatile compounds among 0 h, 8 h, and pw. (**f**,**g**) Volcano plot analysis of the key volatile compounds between (**f**) 0 h and 8 h, (**g**) pw and 8 h. The red dots represent upregulated metabolites, the blue dots represent downregulated metabolites, and the gray dots represent metabolites detected, but they are not significantly different.

The impact of UV-C irradiation on the aroma profile of Pu’er raw tea was systematically analyzed by examining changes in volatile compounds under different treatment durations. The exposure of Pu’er raw tea to UV-C irradiation resulted in significant alterations in its chemical composition, particularly in the aroma compounds that contribute to its characteristic fragrance (Figure 2a). Fresh Pu’er raw tea, which is typically dominated by aldehydes and ketones associated with green and floral notes, underwent a marked transformation as the duration of UV-C treatment increased. The initial fresh and slightly astringent aroma of the untreated tea gradually evolved into a more complex and mature fragrance profile, characterized by a reduction in freshness-associated volatiles and a corresponding increase in compounds such as alcohols and terpenes, which are known for their role in creating the deep, rich floral and fruity notes that are highly valued in aged Pu’er raw tea [21]. This progression from a simpler, more direct aroma to a layered and sophisticated fragrance suggests that UV-C irradiation effectively simulates the chemical changes that naturally occur during the long-term aging process, but within a much shorter time frame. As the duration of UV-C exposure increased, the chemical differentiation among the tea samples became more pronounced, with the 8 h UV-C treated tea showing the closest resemblance to the naturally aged control group (Figure 2b). This indicates that the 8 h treatment is particularly effective in inducing the types of chemical transformations that are essential for developing the desired sensory qualities of aged Pu’er raw tea [22]. The aroma profile of the 8 h UV-C treated tea was found to be complex and multi-dimensional, with a well-balanced combination of floral, fruity, and earthy notes that are typically associated with high-quality aged Pu’er raw tea [23]. The presence of these desirable aromas in the 8 h treated sample indicates that UV-C irradiation accelerates the natural aging process by triggering specific chemical reactions within the tea leaves. The UV-C treatment appears to expedite these reactions, thereby reducing the time required to achieve the desired aging effects from years to mere hours.

The analysis of key volatile compounds in Pu’er raw tea subjected to varying durations of UV-C irradiation was initiated with orthogonal partial least squares discriminant analysis (OPLS-DA) to evaluate the differentiation of the samples based on their chemical profiles (Figure 2c) [24]. The OPLS-DA results demonstrated a clear separation among the tea samples, underscoring the significant impact of UV-C treatment duration on the tea’s chemical composition. This differentiation is vital for elucidating how UV-C irradiation modulates the volatile compounds responsible for the tea’s aroma profile. Subsequently, a heatmap with hierarchical clustering was employed to visualize the relative abundance of these key volatile compounds across different UV-C treatment durations (Figure 2d). The heatmap revealed specific compounds that either increased or decreased in concentration due to UV-C exposure, thereby providing deeper insight into the chemical alterations induced by irradiation. The influence of UV-C irradiation on aroma compounds extended beyond mere oxidation and polymerization processes [25]. The observed increase in alcohols and terpenes, which are pivotal for imparting floral and fruity notes to Pu’er raw tea [26], suggests that UV-C exposure may enhance the biosynthesis of these compounds [27]. Terpenes like linalool and geraniol, known for their pleasant floral aromas, are typically more concentrated in aged teas [28,29]. These compounds generally form through enzymatic reactions during the natural aging process, yet the UV-C treatment appears to accelerate their production, potentially by modulating enzyme activity associated with their biosynthesis [30]. This acceleration, coupled with the enhanced oxidation of polyphenols, culminates in a more mature aroma profile that closely mimics naturally aged Pu’er raw tea [31]. However, it is crucial to recognize that while an 8 h UV-C treatment effectively simulates the natural aging process, extending the treatment beyond this optimal duration may result in overprocessing. The data indicate that prolonged UV-C exposure could lead to a decline in the concentration of essential aroma compounds, such as specific alcohols and terpenes, which may adversely affect the tea’s sensory qualities. This decline is likely attributable to the excessive oxidation and degradation of these compounds under extended UV-C exposure, which could flatten the tea’s aroma profile and diminish the complexity characteristic of high-quality aged tea [32].

Based on the results of PCA and OPLS-DA analyses, the 8 h UV-C treated group exhibited the greatest similarity to the naturally aged sample. The volatile compounds of fresh Pu’er raw tea, UV-C treated for 8 h, and naturally aged sample were further analyzed. The Venn diagram showed that fresh Pu’er raw tea had the fewest types of aroma compounds without aroma compounds unique to the 0 h sample. This indicates that both natural aging and rapid aging treatments significantly enriched the aroma composition compared to fresh Pu’er raw tea (Figure 2e). Furthermore, there were as many as 29 identical volatile compounds between the 8h treatment group and the naturally aged group, accounting for 82.8% and 80.5% of their respective aroma compositions, further illustrating the similarity between the 8h treatment group and the naturally aged sample. Volcano plot analysis revealed that 8h of UV-C irradiation significantly upregulated the levels of key volatile compounds such as Linalool, geraniol, alpha-Terpineol, and 2-Furanmethanol (Figure 2f). Linalool and geraniol contribute floral notes such as rose and lavender, while alpha-Terpineol offers a fresher woody aroma [33]. 2-Furanmethanol, on the other hand, contributes sweet, almond-like notes [34]. Comparing the naturally aged samples and the 8h UV-C irradiation group, the 8h treatment group was found to contain higher levels of alpha-Cadinol and 1,5,7-Octatrien-3-ol, 3,7-dimethyl (Figure 2g). The naturally aged group, on the other hand, was enriched in Cedrol, 2-Cyclohexen-1-one, 5-methyl-2-(1-methylethyl), and 3-Buten-2-one, 4-(2,2,6-trimethyl-7-oxabicyclo[4.1.0]hept-1-yl). Both alpha-Cadinol and Cedrol are sesquiterpenoids, composed of 15 carbon atoms, and both possess woody aromas. Cedrol is typically described as having a cedarwood scent, while alpha-Cadinol may exhibit more complex woody, spicy, or herbaceous aromas [35].

### 3.3. Evaluation of Key Volatile Compounds in Pu’er Raw Tea Under Varying UV-C Treatment Durations

**Figure 3:**
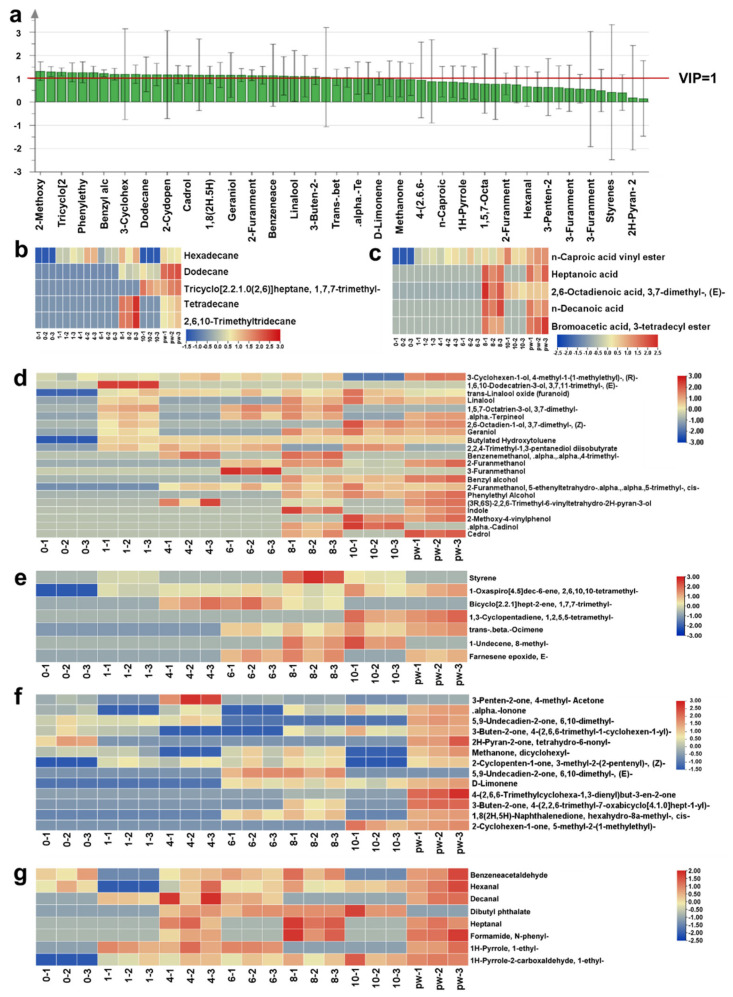
Evaluation of key volatile compounds in Pu’er raw tea under different UV-C treatment durations. (**a**) VIP evaluation of major volatile compounds. (**b**–**g**) Relative abundance and changes in the UV-C irradiation process of (**b**) alkanes, (**c**) fatty acids, (**d**) alcohols, (**e**) terpenes, (**f**) ketones, and (**g**) other compounds, including pyridine, phenols, and nitrogen-containing compounds.

The evaluation of key volatile compounds in Pu’er raw tea treated with varying durations of UV-C irradiation revealed significant changes in the tea’s chemical composition, particularly in compounds critical to its aroma profile. These compounds, including aldehydes, alcohols, ketones, and terpenes, are pivotal in defining the tea’s sensory attributes, influencing its aroma, flavor, and overall consumer acceptance. The study assessed the variation in these compounds under different UV-C treatment durations, offering valuable insights into how UV-C irradiation can accelerate the aging process of Pu’er raw tea, effectively enhancing its desirable qualities [27]. The analysis utilized Variable Importance in Projection (VIP) scores to identify key volatile compounds that significantly differentiated the aroma profiles of tea samples subjected to various UV-C irradiation durations (Figure 3a) [21]. Compounds such as 2-methoxy-4-vinylphenol, phenylethyl alcohol, geraniol, and linalool exhibited high VIP scores, highlighting their substantial contribution to the tea’s floral and sweet notes, which are essential for the aromatic qualities of aged Pu’er raw tea. The prominence of these compounds in the 8 h treated samples suggests this duration as optimal for developing a rich and complex aroma profile that closely resembles naturally aged tea. A significant decrease in aldehyde compounds, such as hexanal and benzeneacetaldehyde, was observed with increasing UV-C exposure, indicative of a reduction in fresh and green notes typically associated with younger teas (Figure 3b) [21]. This reduction aligns with the aging process, where the freshness of the tea diminishes, giving way to more complex and mature aromas. In contrast, alcohol compounds like linalool and phenylethyl alcohol increased significantly with prolonged UV-C treatment, contributing to the development of the highly valued floral and fruity notes in aged Pu’er raw tea (Figure 3c) [22]. These alcohols, particularly linalool, are critical in creating the floral and sweet character of aged teas, and their increased presence suggests that UV-C treatment accelerates their synthesis or stabilizes them against degradation. The concentration of key ketone compounds, such as β-ionone and geranylacetone, also increased, adding depth and richness to the tea’s aroma profile (Figure 3d) [36]. These ketones, associated with woody, violet-like, fruity, and floral nuances, enhance the complex and layered aromas characteristic of aged teas. The increase in these compounds suggests that UV-C irradiation not only accelerates the aging process but also enhances the development of these desirable aroma characteristics by facilitating the breakdown of larger terpenoid precursors under UV-C light. The analysis of terpene compounds revealed a marked increase in their concentration after 8 h of UV-C treatment, indicating this duration as optimal for developing the nuanced and layered aroma profile characteristic of aged Pu’er raw tea (Figure 3e). Terpenes such as limonene and α-terpineol, which contribute to the tea’s citrusy and pine-like notes, were particularly enhanced, adding brightness and complexity to the tea’s aroma. However, extending UV-C treatment beyond 8 h could lead to a plateau or decline in these compounds’ concentration, potentially diminishing the overall quality of the tea’s aroma (Figure 3f,g). This plateau effect may result from the saturation of the reactions responsible for terpene synthesis or from degradation processes that become dominant with prolonged UV-C exposure. In conclusion, UV-C irradiation, particularly for durations of up to 8 h, effectively simulates the natural aging process of Pu’er raw tea by enhancing the concentration of key volatile compounds. These compounds, including aldehydes, alcohols, ketones, and terpenes, are crucial in defining the tea’s aromatic profile. The findings suggest that 8 h of UV-C treatment is optimal for balancing the acceleration of the aging process with the maintenance of the tea’s complexity and richness. Prolonged exposure beyond this point may lead to overprocessing, compromising the desired sensory qualities of the tea. This method offers a promising approach for producing high-quality aged Pu’er raw tea in a shorter time frame, potentially reducing costs and making such teas more accessible to a broader market. Future research should focus on the long-term stability of these UV-C-induced changes and consumer acceptance to provide further insights into the commercial potential of this technique. These findings also suggest a broader applicability of UV-C irradiation in the accelerated aging of other tea types, where similar aromatic characteristics are desired, emphasizing the need for precise control over irradiation conditions to optimize the final product quality in the tea industry.

### 3.4. Analysis of the Impact of UV-C Treatment Duration on the Chemical Composition and Sensory Characteristics of Pu’er Raw Tea

**Figure 4:**
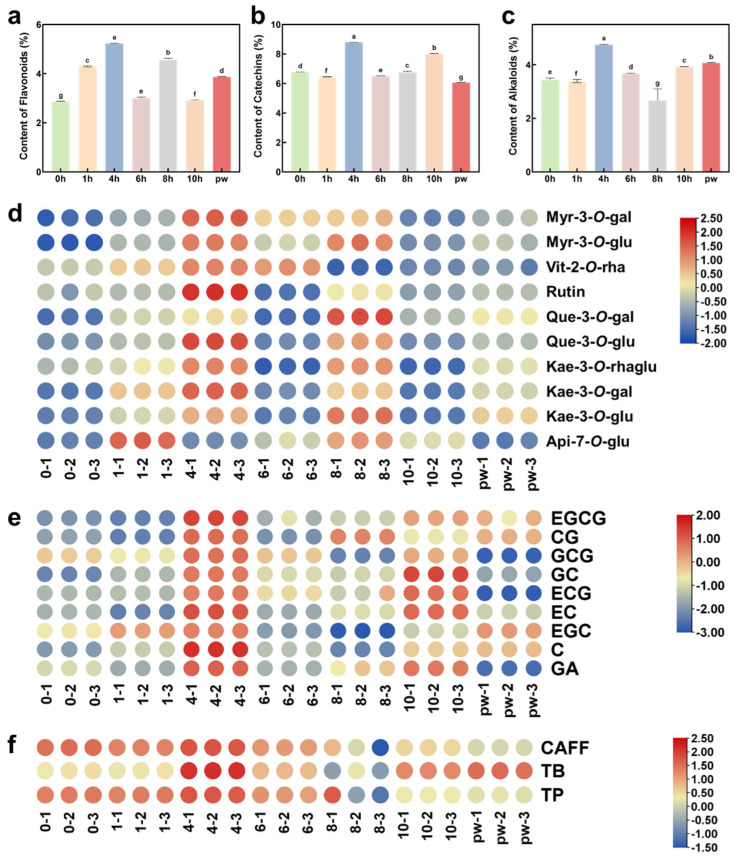
Analysis of the impact of UV-C treatment duration on the chemical composition and sensory characteristics of Pu’er raw tea. (**a**) Total flavonoid content measured in Pu’er raw tea under different UV-C irradiation durations. (**b**) Total catechins content measured by HPLC. (**c**) Total alkaloid content measured by HPLC. (**d**) Heatmap showing the relative abundance of flavonoids. (**e**) Heatmap showing the relative abundance of catechins. (**f**) Heatmap showing the relative abundance of alkaloids. Groups without any same letters labeled were significantly different (*p* < 0.05).

The HPLC analysis revealed significant changes in the chemical composition of Pu’er raw tea subjected to UV-C irradiation for different durations. These changes are reflected in the levels of flavonoids, catechins, and alkaloids, which are closely related to the overall quality and sensory properties of the tea (Figure 4a–c) [37]. Freshly processed Pu’er raw tea exhibited higher levels of catechins, leading to a relatively astringent and bitter taste in the tea infusion. In contrast, naturally aged Pu’er raw tea showed a reduction in catechin levels and an increase in flavonoid content, resulting in a smoother and more mellow sensory profile (Figure 4a,b) [38]. At the same time, the reduction in alkaloid content, particularly caffeine, significantly improved the sensory quality of the tea, as lower caffeine levels contribute to a smoother taste and less bitterness (Figure 4c) [39]. Heatmap analysis further supports these findings (Figure 4d–f), showing that the relative abundance of catechins such as EGCG in naturally aged tea is lower than in fresh tea, while the relative abundance of flavonoids like quercetin and kaempferol gradually increase [40]. This indicates that UV-C irradiation significantly affects the chemical composition of Pu’er raw tea [41]. Significant changes were observed as early as after 2 h of exposure. At this early stage, the increase in catechin levels may be due to UV-C irradiation promoting enzyme activity and enhancing biosynthesis [42], which in turn increases the astringency and bitterness of the tea, as EGCG and ECG are known contributors to these sensory characteristics. As the duration of UV-C irradiation was extended to 4–6 h, the chemical composition began to change, with a decrease in catechin levels and a gradual increase in flavonoid levels. This suggests that UV-C irradiation accelerates the oxidation process, effectively simulating the natural aging process within a shorter time frame. The heatmap also indicates that at the 6 h mark, the chemical composition of the samples began to resemble that of naturally aged tea, particularly in terms of flavonoid and catechin content. By the 8 h mark, the chemical composition of UV-C treated tea closely resembled that of naturally aged tea (pw). At this point, the catechin content was significantly reduced, while the content of flavonoids increased. Additionally, the content of caffeine decreased and theobromine increased, resulting in a smoother, more refined, and richer flavor profile similar to traditionally aged Pu’er raw tea [43]. The heatmap further indicates that the relative abundance of key compounds such as catechins, flavonoids, and alkaloids in the 8 h treated tea was very close to that of naturally aged tea, suggesting that this duration of UV-C treatment is optimal for simulating natural aging. The chemical composition of the 8 h UV-C treated tea and naturally aged tea exhibited significant similarities, particularly in the reduction in catechin levels and the increase in flavonoid levels. This balance of compounds contributes to the formation of desirable flavor characteristics—lower catechin levels reduce astringency, while the increased flavonoid content enriches the tea’s complex aroma and flavor, particularly enhancing floral and fruity notes that are important for the sensory quality of the tea. This similarity suggests that 8 h of UV-C treatment can effectively replicate the effects of several years of natural aging, offering a feasible method for producing high-quality aged Pu’er raw tea in a much shorter time frame. These findings indicate that UV-C irradiation could be a promising method for accelerating the aging process of Pu’er raw tea, providing significant benefits in terms of production efficiency and market expansion. Future research should focus on the long-term stability of UV-C treated tea and the effects of UV-C treatment on the accelerated aging of other long-term storage teas, such as white tea.

## 4. Conclusions

This study investigated the effects of UV-C irradiation on the quality of Pu’er raw tea, aiming to establish an efficient model for its rapid aging. The experimental results demonstrated that UV-C irradiation significantly accelerated the aging process of Pu’er raw tea within 8 h. In terms of sensory quality, the UV-C treated tea exhibited reduced astringency compared to untreated fresh tea, gradually developing a rich and smooth taste profile characterized by complex aromas of aged notes, floral, and sweet fragrances as the irradiation time increased. GC-MS analysis revealed a significant increase in the content of aldehydes and terpenes, which are closely associated with the tea’s aroma. Notably, compounds such as linalool and geraniol, known for their pleasant floral scents, contributed to the enhanced depth and richness of the tea’s aroma. Further HPLC analysis indicated that with prolonged UV-C exposure, the levels of catechins and caffeine significantly decreased, while the concentration of flavonoids increased. These findings provide strong evidence for the changes in the tea’s aroma and flavor profile induced by UV-C irradiation. Notably, the tea samples treated with UV-C for 8 h closely resembled naturally aged Pu’er raw tea in both sensory description and chemical composition. This discovery offers a novel approach to the rapid aging of Pu’er raw tea, as well as other storage-resistant teas.

## Figures and Tables

**Table 1:** Sensory evaluation of different Pu’er raw tea samples.

Sample	Appearance	Infusion Color	Aroma	Taste	Infused Leaf
0 h	Uniform, tight, neat surface, clear veins, greenish bright	Light yellowish-orange, bright	Strong aroma, fresh and refreshing	Thick, strong, brisk	Tender, with buds, red edges, yellowish-green
1 h	Tight, neat surface, clear veins, more veins, greenish bright	Light yellowish-orange, bright	Fresh aroma, tender aroma, refreshing	Thick, fresh, brisk	Tender, with buds, red edges, yellowish-green
4 h	Tight, neat surface, clear veins, more veins, greenish-yellow	Yellowish-orange, bright	Fresh aroma, tender aroma	Thick, brisk, mellow	Tender, with buds, red edges, yellowish-green
6 h	Tight, neat surface, slightly golden, yellowish	Orange, bright	Ripe aroma, mellow	Fermented, refreshing with floral aroma	Tender, with buds, red edges, yellowish-green
8 h	Tight, neat surface, golden, yellowish	Light yellowish-orange, bright	Ripe aroma, faint aroma	Fermented, refreshing with floral aroma, faintly brisk	Tender, with buds, red edges, yellowish-green
10 h	Tight, neat surface, golden, dark yellow	Light yellowish-orange, bright	Ripe aroma, faint aroma	Fermented, faintly brisk	Tender, with buds, red edges, more red edges, yellowish-green
PW	Uniform, tight, neat surface, golden veins, yellowish	Yellowish-orange, bright	Strong aroma, floral aroma	Fermented, faintly brisk, with floral aroma	Tender, with buds, red edges, more red edges, yellowish-green

## Data Availability

The raw data supporting the conclusions of this article will be made available by the authors on request.

## References

[1] Wang C., Li J., Wu X., Zhang Y., He Z., Zhang Y., Zhang X., Li Q., Huang J., Liu Z. (2022). Pu-erh tea unique aroma: Volatile components, evaluation methods and metabolic mechanism of key odor-active compounds. Trends Food Sci. Technol..

[2] (2008). Product of geographical indication - Puer tea.

[3] Ahmed S., Stepp J.R. (2025). Pu-erh tea: Botany, ethnobotany, production, and chemistry. Tea in Health and Disease Prevention.

[4] Zhu R., Chen Z., Lv H., Pan Y., Feng X., Chen G., Hu W., Xu T., Fan F., Gong S. (2023). Another thread to uncover the aging mystery of white tea: Focusing on the natural nanoparticles in tea infusion. Food Chem..

[5] Lv H., Feng X., Song H., Ma S., Hao Z., Hu H., Yang Y., Pan Y., Zhou S., Fan F. (2023). Tea storage: A not thoroughly recognized and precisely designed process. Trends Food Sci. Technol..

[6] Qi D., Miao A., Cao J., Wang W., Chen W., Pang S., He X., Ma C. (2018). Study on the effects of rapid aging technology on the aroma quality of white tea using GC–MS combined with chemometrics: In comparison with natural aged and fresh white tea. Food Chem..

[7] Wang S., Li Y., Qu Y., Guo D., Luo S., Wang J., Peng C., Zhang X., Jiang H. (2023). Enhancing effects of 60Co irradiation on the extraction and activities of phenolic components in edible Citri Sarcodactylis Fructus. Food Chem..

[8] Chen T., Peng W., Zhao Y., Liu Y., Wang B. (2016). Research on aging effect of unfermented Pu’er tea by high-voltage pulsed electric field. Agric. Res..

[9] Zhao Y., Zuo J., Yuan S., Shi W., Shi J., Feng B., Wang Q. (2021). UV-C Treatment Maintains the Sensory Quality, Antioxidant Activity and Flavor of Pepino Fruit during Postharvest Storage. Foods.

[10] Pombo M.A., Rosli H.G., Martínez G.A., Civello P.M. (2011). UV-C treatment affects the expression and activity of defense genes in strawberry fruit (Fragaria× ananassa, Duch.). Postharvest Biol. Technol..

[11] Pan J., Vicente A.R., Martínez G.A., Chaves A.R., Civello P.M. (2004). Combined use of UV-C irradiation and heat treatment to improve postharvest life of strawberry fruit. J. Sci. Food Agric..

[12] Vergne M.J., Patras A., Bhullar M.S., Shade L.M., Sasges M., Rakariyatham K., Pan C., Xiao H. (2018). UV-C irradiation on the quality of green tea: Effect on catechins, antioxidant activity, and cytotoxicity. J. Food Sci..

[13] Pan Y., Lv H., Feng X., Zhou S., Hu H., Chen S., Cheng Y., Fan F., Gong S., Chen P. (2023). Epigallocatechin gallate (EGCG) alleviates the inflammatory response and recovers oral microbiota in acetic acid-induced oral inflammation mice. Food Funct..

[14] (2018). Methodology for sensory evaluation of tea.

[15] Ströhla L.C., Hidangmayum K.S., Waehrens S.S., Orlien V., Petersen M.A. (2022). Effect of processing and accelerated storage on the volatile composition and sensory profile of a tomato soup. Food Qual. Saf..

[16] Guo H., Pan Y., Li C., Fu Y., Cao Y., Chu Q., Chen P. (2024). Influence of Various Tea Utensils on Sensory and Chemical Quality of Different Teas. Plants.

[17] Lu L., Zhang J., Wu F., Xie G., Shan Z., Liu X. (2022). Flavor profile variations of Huangjiu brewed in different traditional Chinese solar terms. Food Qual. Saf..

[18] Pan Y., Feng X., Zhou S., Yang S., Qiu P., Gong S., Chu Q., Chen P. (2023). Hydroxyls on the B ring and gallic acyl are essential for catechins to restrain ADP-induced thrombosis. Food Funct..

[19] Kang Y., Li C., Li H., Li J., Jiang K. (2022). Differentiation of qualified tea beverages from spoiled ones by the LC-MS–based analysis of their polycarboxylic acids. Food Qual. Saf..

[20] Elnour A.A.M., Mirghani M.E.S., Kabbashi N.A., Hamid Musa K., Shahabipour F., Ashammakhi N., Hamid Abdurahman N. (2022). Comparative study of the characterisation and extraction techniques of polyphenolic compounds from Acacia seyal gum. Food Qual. Saf..

[21] Golombek P., Wacker M., Buck N., Durner D. (2021). Impact of UV-C treatment and thermal pasteurization of grape must on sensory characteristics and volatiles of must and resulting wines. Food Chem..

[22] Mandal R., Wiktor A., Mohammadi X., Pratap-Singh A. (2022). Pulsed UV light irradiation processing of black tea infusions: Effect on color, phenolic content, and antioxidant capacity. Food Bioprocess Technol..

[23] Jia W., Rajani C., Lv A., Fan T.-P., Zheng X. (2022). Pu-erh tea: A review of a healthful brew. J. Tradit. Chin. Med. Sci..

[24] Bylesjö M., Rantalainen M., Cloarec O., Nicholson J.K., Holmes E., Trygg J. (2006). OPLS discriminant analysis: Combining the strengths of PLS-DA and SIMCA classification. J. Chemom..

[25] Collings E.R., Alamar M.C., Márquez M.B., Kourmpetli S., Kevei Z., Thompson A.J., Mohareb F., Terry L.A. (2021). Improving the Tea Withering Process Using Ethylene or UV-C. J. Agric. Food Chem..

[26] Pripdeevech P., Wongpornchai S. (2013). Odor and Flavor Volatiles of Different Types of Tea.

[27] Wang X., Cao J., Cheng X., Liu X., Zhu W., Li Y., Wan X., Chen S., Liu L. (2024). UV-B application during the aeration process improves the aroma characteristics of oolong tea. Food Chem..

[28] Wang H., You X.-Q., Chen Z. (2002). The chemistry of tea volatiles. Tea: Bioactivity and Therapeutic Potential.

[29] Guo X., Schwab W., Ho C.-T., Song C., Wan X. (2022). Characterization of the aroma profiles of oolong tea made from three tea cultivars by both GC–MS and GC-IMS. Food Chem..

[30] Hu J., Feng X., Song H., Hao Z., Ma S., Hu H., Yang Y., Zhou S., Pan Y., Fan F. (2024). Enzymatic reactions throughout cultivation, processing, storage and post-processing: Progressive sculpture of tea quality. Trends Food Sci. Technol..

[31] Zhang X.-Y., Zhao Y., Qian Y., Leng Y., Wang B.-J. (2020). Effect of high voltage pulsed electric field on aroma and aging time of unfermented Pu’er tea. Shipin Kexue/Food Sci..

[32] Xu S., Zeng X., Wu H., Shen S., Yang X., Deng W.-W., Ning J. (2021). Characterizing volatile metabolites in raw Pu’er tea stored in wet-hot or dry-cold environments by performing metabolomic analysis and using the molecular sensory science approach. Food Chem..

[33] Liang Y., Wang Z., Zhang L., Dai H., Wu W., Zheng Z., Lin F., Xu J., Huang Y., Sun W. (2024). Characterization of volatile compounds and identification of key aroma compounds in different aroma types of Rougui Wuyi rock tea. Food Chem..

[34] Mei S., Cao Y., Zhang G., Zhou S., Wang Y., Gong S., Chu Q., Chen P. (2022). Construction of Sensory/Mass Spectrometry Feedback Platform for Seeking Aroma Contributors during the Aroma Enhancement of Congou Black Tea. Plants.

[35] Chang S.-T., Cheng S.-S., Wang S.-Y. (2001). Antitermitic activity of essential oils and components from taiwania (*Taiwania cryptomerioides*). J. Chem. Ecol..

[36] Gök S.B., Yıkmıs S., Levent O., Karatas S. (2022). Impact of UV-C and thermal pasteurization on bioactive compounds, sensory characteristics and aroma profile of traditionally produced koruk vinegar. J. Food Saf. Food Qual..

[37] Jiang Z., Han Z., Zhu M., Wan X., Zhang L. (2023). Effects of thermal processing on transformation of polyphenols and flavor quality. Curr. Opin. Food Sci..

[38] Wang S., Qiu Y., Gan R.-Y., Zhu F. (2022). Chemical constituents and biological properties of Pu-erh tea. Food Res. Int..

[39] Zhang L., Cao Q.-Q., Granato D., Xu Y.-Q., Ho C.-T. (2020). Association between chemistry and taste of tea: A review. Trends Food Sci. Technol..

[40] Zhang J.-W., Shen X.-M., Zhao Y.-F., Wang W., Zhang Q., Zhou Y.-F., Li J.-H. (2021). Changes in content of three main flavonoids in raw Pu-erh tea with different storage years. Southwest China J. Agric. Sci..

[41] Pedan V., Rohn S., Holinger M., Hühn T., Chetschik I. (2018). Bioactive compound fingerprint analysis of aged raw Pu’er tea and young ripened Pu’er tea. Molecules.

[42] Ozturk B., Seyhan F., Ozdemir I.S., Karadeniz B., Bahar B., Ertas E., Ilgaz S. (2016). Change of enzyme activity and quality during the processing of Turkish green tea. LWT.

[43] Ge Y., Li N., Fu Y., Yu X., Xiao Y., Tang Z., Xiao J., Wu J.-L., Jiang Z.-H. (2021). Deciphering superior quality of Pu-erh tea from thousands of years’ old trees based on the chemical profile. Food Chem..

